# Amplifications of *EVX2* and *HOXD9-HOXD13* on 2q31 in mature cystic teratomas of the ovary identified by array comparative genomic hybridization may explain teratoma characteristics in chondrogenesis and osteogenesis

**DOI:** 10.1186/s13048-024-01458-5

**Published:** 2024-06-21

**Authors:** Wen-Chung Wang, Tai-Cheng Hou, Chen-Yun Kuo, Yen-Chein Lai

**Affiliations:** 1 Department of Obstetrics and Gynecology, Jen-Ai Hospital, Taichung, Taiwan; 2 Department of Pathology, Jen-Ai Hospital, Taichung, Taiwan; 3 Department of Medical Laboratory and Biotechnology, Chung Shan Medical University, No.110, Sec. 1, Chien Kuo N. Road, Taichung, 402 Taiwan, R.O.C.; 4 Clinical Laboratory, Chung Shan Medical University Hospital, Taichung, Taiwan

**Keywords:** Mature cystic teratoma of the ovary, Array comparative genomic hybridization, Chondrogenesis, Osteogenesis

## Abstract

**Background:**

Teratomas are a common type of germ cell tumor. However, only a few reports on their genomic constitution have been published. The study of teratomas may provide a better understanding of their stepwise differentiation processes and molecular bases, which could prove useful for the development of tissue-engineering technologies.

**Methods:**

In the present study, we analyzed the copy number aberrations of nine ovarian mature cystic teratomas using array comparative genomic hybridization in an attempt to reveal their genomic aberrations.

**Results:**

The many chromosomal aberrations observed on array comparative genomic hybridization analysis reveal the complex genetics of this tumor. Amplifications and deletions of large DNA fragments were observed in some samples, while amplifications of *EVX2* and *HOXD9-HOXD13* on 2q31.1, *NDUFV1* on 11q13.2, and *RPL10, SNORA70, DNASE1L1, TAZ, ATP6AP1*, and *GDI1* on Xq28 were found in all nine mature cystic teratomas.

**Conclusions:**

Our results indicated that amplifications of these genes may play an important etiological role in teratoma formation. Moreover, amplifications of *EVX2* and *HOXD9-HOXD13* on 2q31.1, found on array comparative genomic hybridization, may help to explain the characteristics of teratomas in chondrogenesis and osteogenesis.

**Supplementary Information:**

The online version contains supplementary material available at 10.1186/s13048-024-01458-5.

## Abstract (summary)

Nine ovarian mature cystic teratomas were analyzed by aCGH. The amplifications of *EVX2* and *HOXD9-HOXD13* on 2q31.1 can be used to explain the characteristics of teratomas in chondrogenesis and osteogenesis.

**Supplementary Information:**

The online version contains supplementary material available at 10.1186/s13048-024-01458-5.

## Background

Ovarian germ cell tumors account for 15–20% of all ovarian malignancies and the incidence of malignant ovarian germ cell tumors is 2–6% [1]. The vast majority are teratomas [2]. There are several types of teratomas: mature cystic, immature, and monodermal. Among them, 97% are cystic mature teratomas, which are also called dermoid cysts [3]. They occur in females at almost any age, but most commonly between the ages of 20 and 30 [4]. The genetic and environmental conditions that confer teratoma development remain poorly understood [5]. Further understanding of teratoma polyderm differentiation promises to provide insight into disorders of ovarian and germ cell lineages, such as ovarian tumor formation and infertility [5].

In light of the ethical issues surrounding the use of human stem cells, teratomas are being looked at as an alternative for research studies, as they lack the potential to grow into functional human beings. Formation of three somatic germ layers within teratomas is considered the best indicator of the pluripotency of human embryonic stem cell lines [6, 7]. A further understanding of this process should aid in the development of safer human embryonic stem cell therapies and elucidation of the principles of teratoma formation [8]. In addition, teratomas represent an alternative development model, as developmental processes cannot be investigated in intact mammalian embryos [9]. Teratomas exhibit arrangements of different tissue types that in many ways recapitulate organogenesis within the embryo [10]. Therefore, studying teratomas could result in a better understanding of their stepwise developmental processes and molecular bases, as well as useful information for the development of tissue-engineering technologies [11].

Recently, array comparative genomic hybridization (aCGH) technology has been applied to increase our understanding of oncogensis in a variety of cancer types with some success [12]. However, few studies have been conducted on the developmental role of genomic constitution in mature cystic teratomas of the ovary. There have only been a limted number of reports on ovarian germ cell tumors [13, 14]. The purpose of this study is to clarify the key mechanism for differentiation during teratoma formation. We analyzed the copy number aberrations in nine mature cystic teratomas of the ovary from Taiwanese patients, via the application of aCGH technology, in an attempt to understand the neoplastic or developmental nature and molecular pathogenesis of this tumor.

## Materials and methods

### Clinical samples

The Institutional Review Board of Chung Shan Medical University Hospital approved all procedures and informed consent was obtained from all subjects prior to collecting their genetic material for the study (reference number CS2-15060). Nine 20–40 year-old patients with ovarian teratoma were enrolled (Tera-1 to -9), including one with bilateral mature cystic teratomas of the ovaries (Tera-9, right and left (data not shown)) [15, 16]. Data of left Tera-9 is not shown due to similarities with right Tera-9. The baseline characteristics of these mature cystic teratomas of the ovary have been described in detail elsewhere [17]. These tumors were considered to be mature without immature components after examination of multiple sections. There was no evidence of malignancy.

### Isolation of DNA from blood

Genomic DNA was extracted from paraffin-embedded sections of the teratomas with QIAamp Tissue Kit (Qiagen GmbH., Hilden, Germany) according to the manufacturer’s instructions. DNA was taken from solid nodule within the inner site of the tumor and finally dissolved in 100 µl of TE buffer (10 mM Tris-HCl, pH 8.0, and 1 mM EDTA). DNA concentration of each sample was measured using the NanoDrop 2000c spectrophotometer (Thermo Fisher Scientific, Waltham, MA, USA).

### Array comparative genomic hybridization (aCGH) analysis

Samples were screened on 60-K oligonucleotide aCGH at 0.5-Mb resolution, with SurePrint G3 ISCA V2 CGH Microarray Kit (Agilent Technologies, California, USA). Sample and reference genomic DNAs (Promega female) were labelled by random priming using Genomic DNA Enzymatic Labeling Kit (Agilent Technologies, California, USA), and purified according to the manufacturer’s protocol. Labelled sample DNA (400 ng) was co-precipitated with equal volumes of labelled reference DNA (Promega, pooled female) and 2.5 µg/µl human COT-1 DNA. The samples were hybridized to the microarray at 65 °C for 40 h. Scanning and image acquisition were carried out using Agilent microarray scanner D (Agilent Technologies, California, USA). Data analysis was performed using Feature Extraction (FE) software v10.5 (Agilent Technologies, California, USA). Copy number was determined by a conservative log2 ratio threshold. Copy number aberrations from segmented data were based on human genome build GRCh37/UCSC hg19. Amp refers to amplification (gain ≥ 0.15) and Del refers to deletion (loss ≤ − 0.15). Profile deviations consisting of 10 or more neighbouring oligonucleotides were considered genomic aberrations.

### Immunohistochemistry

Sections from 4-micrometer-thick, formalin-fixed, paraffin-embedded mature cystic teratomas of the ovary were used for immunohistochemical analysis. Immunohistochemistry for NDUFV1 was performed using a rabbit antibody after antigen affinity purification (1:150 dilution; Proteintech Group, Rosemont, IL, USA). Antigen retrieval was performed with citrate buffer pH 6.0 at 95 °C for 30 min. Tissue staining was carried out with a DAB substrate chromogen solution. Slides were counterstained with hematoxylin, dehydrated, and mounted. Tumor sections with increase in NDUFV1 protein expression were visualized via light microscopy.

## Results

### Array comparative genomic hybridization (aCGH) analysis

All mature cystic teratomas of the ovary had a normal 46, XX karyotype. We performed analysis twice for Tera-5 and Tera-6 and thrice for Tera-2 to achieve better derivative log ratio spreads (DLRs). DLRs for Tera-1 to Tera-9 were 0.506, 0.220, 0.689, 0.114, 0.200, 0.244, 0.203, 0.352, and 0.245, respectively.

**Fig. 1:**
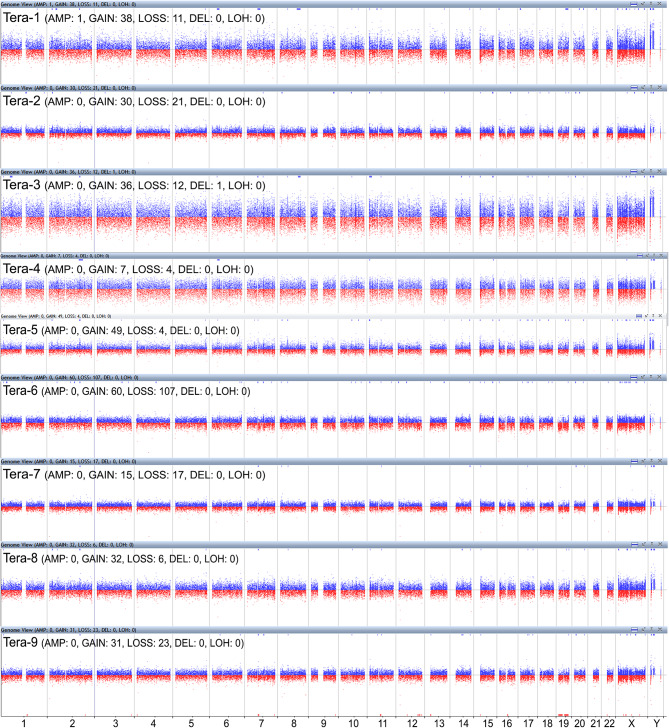
Array comparative genomic hybridization (aCGH) analysis: Whole genomic view (Amp/Del) of aCGH analysis shows pathological genetic imbalances in multiple chromosomes of nine mature cystic teratomas of the ovary from Taiwanese patients

**Fig. 2:**
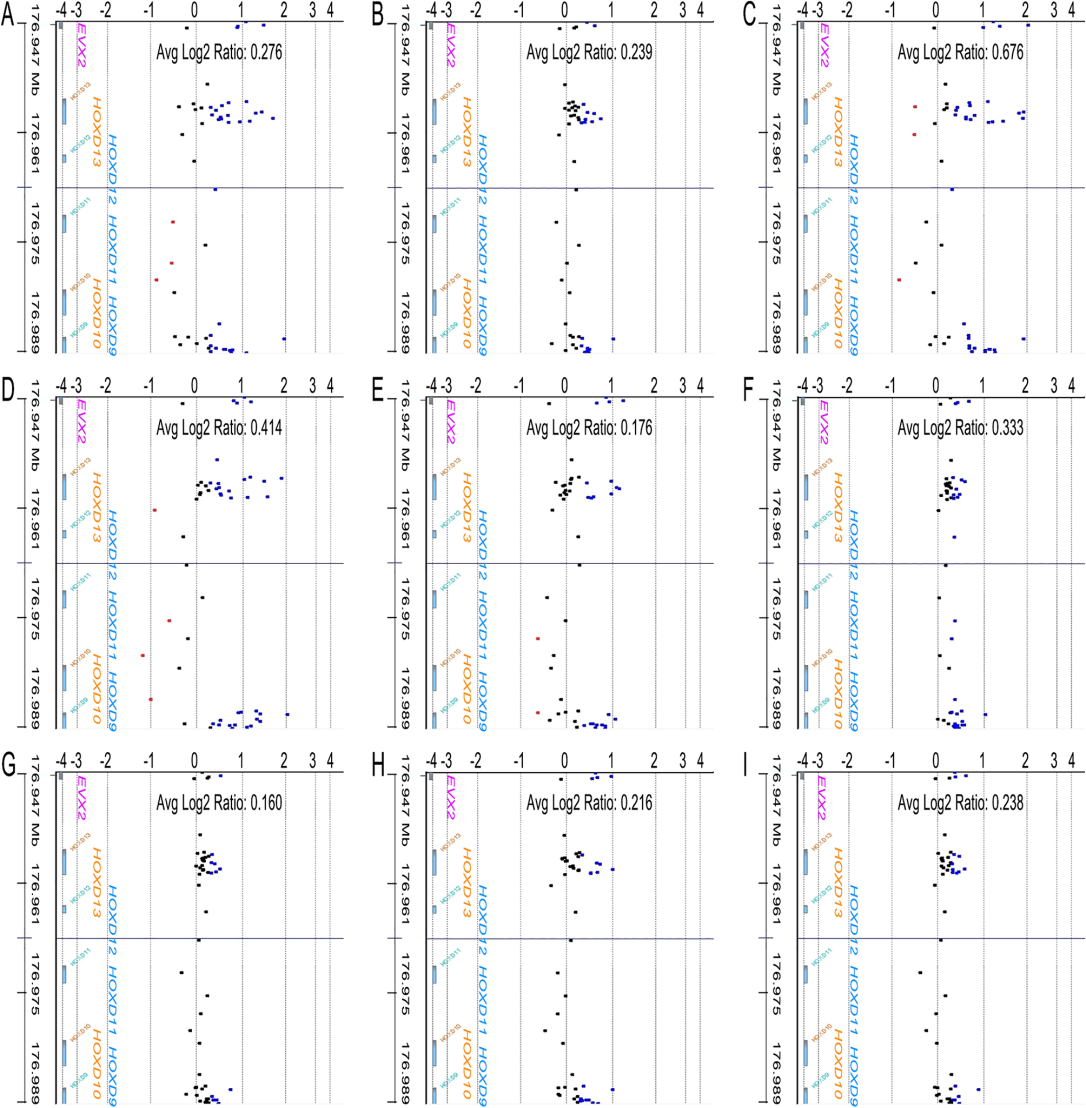
Array comparative genomic hybridization (aCGH) analysis: Gene view (Amp/Del) of aCGH analysis shows amplifications of *EVX2, HOXD13, HOXD12, HOXD11, HOXD10*, and *HOXD9* on 2q31.1, Tera-1 to Tera-9 (**A-I**). X axis: Log2 Ratio; Y axis: 2q31.1 (176.947–176.989 MB)

**Fig. 3:**
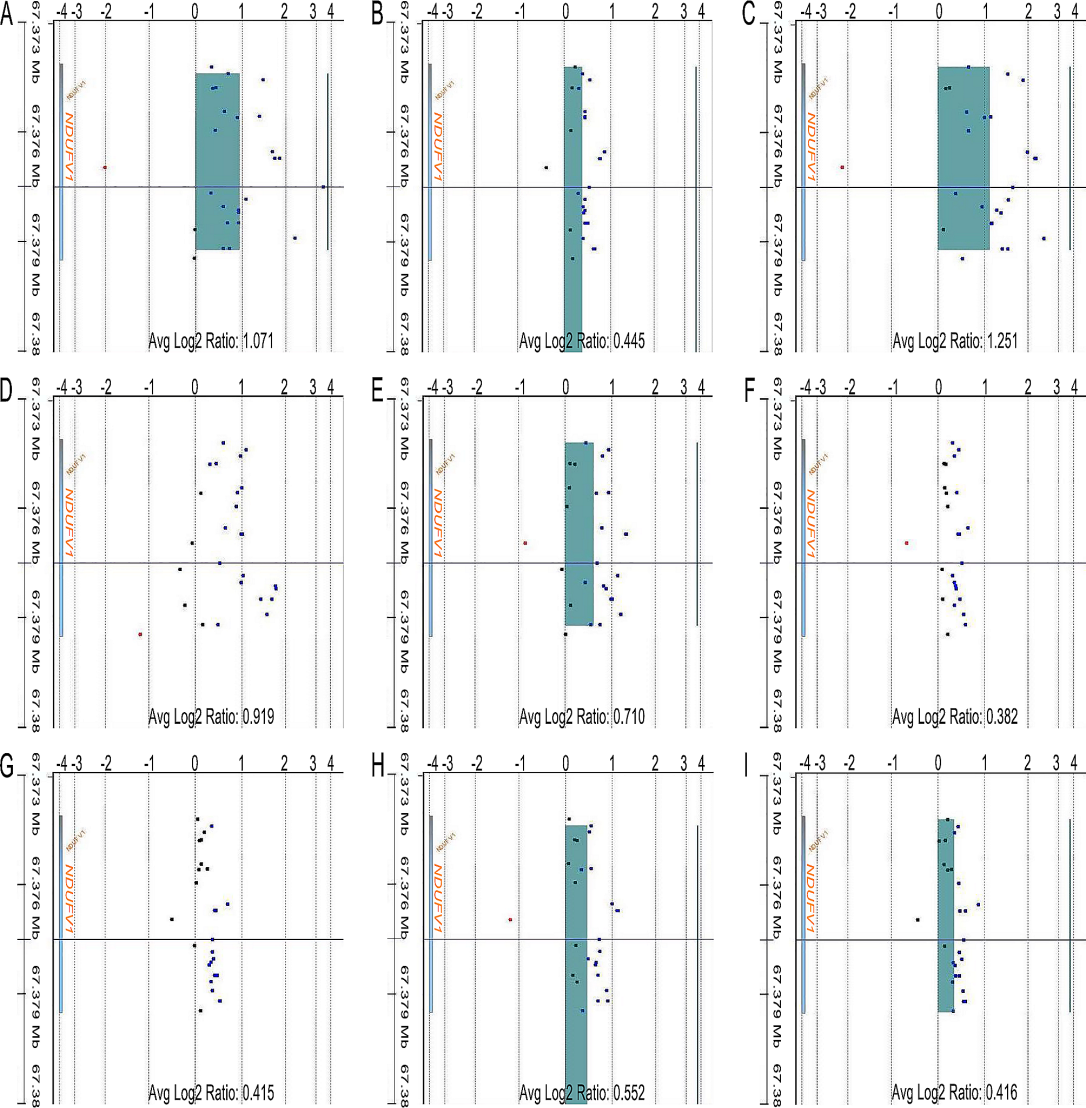
Array comparative genomic hybridization (aCGH) analysis: Gene view (Amp/Del) of aCGH analysis shows amplification of *NDUFV1* on 11q13.2, Tera-1 to Tera-9 (**A-I**). X axis: Log2 Ratio; Y axis: 11q13.2 (67.373–67.382 MB)

**Fig. 4:**
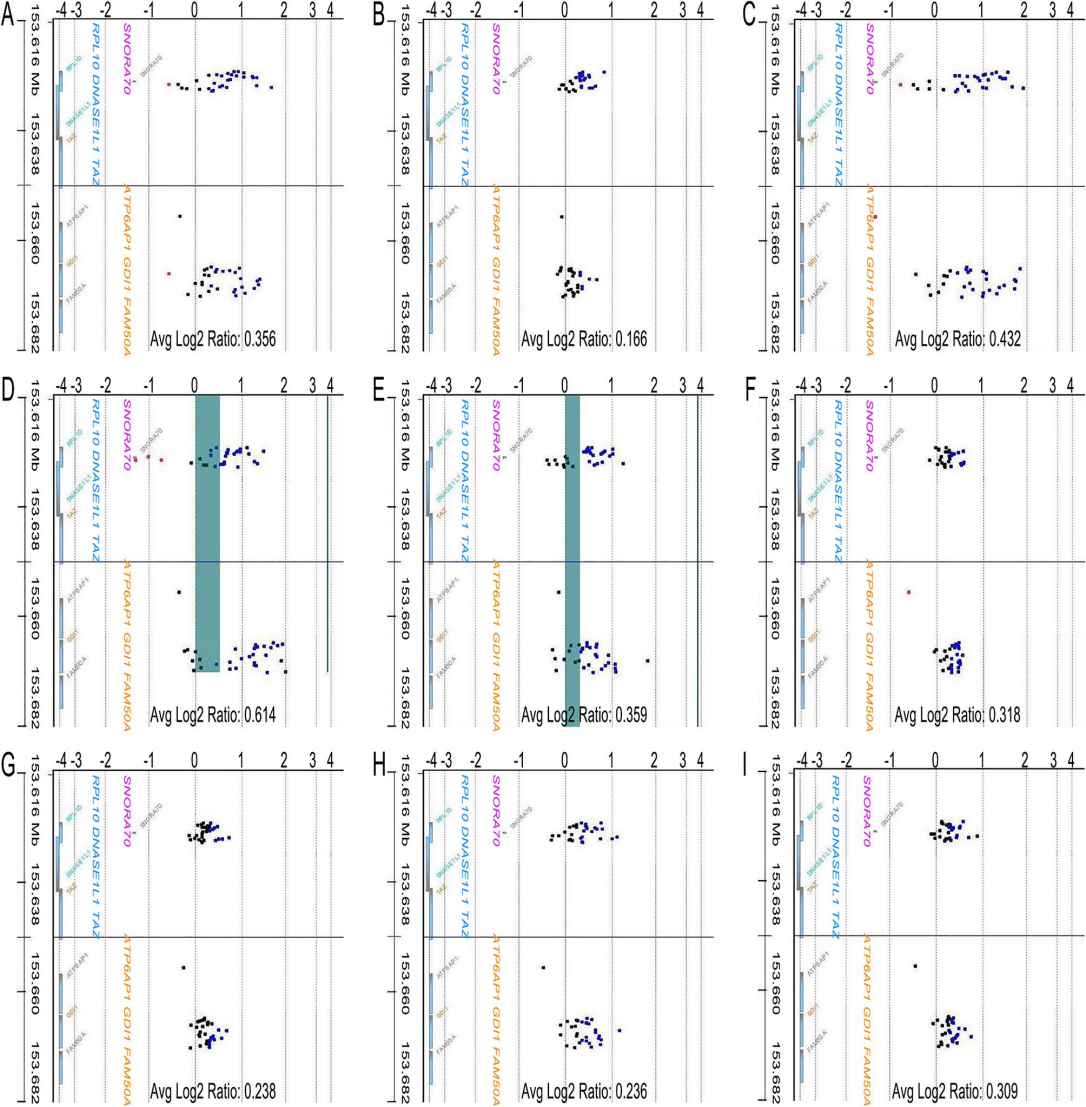
Array comparative genomic hybridization (aCGH) analysis: Gene view (Amp/Del) of aCGH analysis shows gene amplifications on chromosome Xq28, Tera-1 to Tera-9 (**A-I**). Upper panel: *RPL10, SNORA70, DNASE1L1*, and *TAZ*; lower panel: *ATP6AP1, GDI1* and *FAM50A*. X axis: Log2 Ratio; Y axis: Xq28 (153.616-153.682 MB)

Unequal DNA copy numbers were noted in many chromosomal aberrations in all nine samples on aCGH analysis (Fig. 1). Common chromosomal aberrations and their extents are listed in Tables 1 and 2. Amplifications of *EVX2, HOXD13, HOXD12, HOXD11, HOXD10*, and *HOXD9* on a 41.6 kb segment of 2q31.1 (average log2 ratio = 0.303, Fig. 2), *NDUFV1* on a 2.8 kb segment of 11q13.2 (average log2 ratio = 0.685, Fig. 3), and *RPL10, SNORA70, DNASE1L1, TAZ, ATP6AP1*, and *GDI1* on a 44.5 kb segment of chromosome Xq28 (average log2 ratio = 0.336, Fig. 4) were identified in all teratomas. Average log2 ratios for Tera-1 to Tera-9 were 0.276, 0.239, 0.676, 0.414, 0.176, 0.333, 0.160, 0.216, and 0.238, respectively, on the 41.6 kb segment of 2q31.1 (Fig. 2). In addition, average log2 ratios for Tera-1 to Tera-9 were 1.071, 0.445, 1.251, 0.919, 0.710, 0.382, 0.415, 0.552, and 0.416, respectively, on the 2.8 kb segment of 11q13.2 (Figs. 3) and 0.356, 0.166, 0.432, 0.614, 0.359, 0.318, 0.238, 0.236, and 0.309, respectively, on the 44.5 kb segment of chromosome Xq28 (Fig. 4).

**Table 1:** Chromosomal aberrations patterns common to 7 or more teratomas on array comparative genomic hybridization (aCGH) analysis

Cytoband	Location	Size	Gene Names	ISCA Disease	*N* samples
2q31.1	176,947,808∓176,989,433	41,626	*EVX2, HOXD13, HOXD12, HOXD11, HOXD10, HOXD9*	Synpolydactyly,	9
11q13.2	67,376,862∓67,379,713	2,852	*NDUFV1*	Leukodystrophy	9
Xq28	153,627,293∓153,671,782	44,490	*RPL10, SNORA70, DNASE1L1, TAZ, ATP6AP1, GDI1*	X-linked mental retardation	9
10q21.3	64,572,947∓64,575,525	2,579	*EGR2*	Autism	8
14q32.2	101,291,322∓101,291,820	499	*-*	MEG3/NA	8
20q13.32	57,425,665∓57,427,372	1,708	*GNASAS, GNAS*	Albright hereditary osteodystrophy, GNAS_ICRegion/NA	8
Xq13.1	70,460,258∓70,473,291	13,034	*BCYRN1, ZMYM3*	X-linked mental retardation	8
Xq28	153,601,110–153,627,292	26,183	*FLNA, EMD*	X-linked mental retardation	8
2q31.1	172,950,396∓172,953,729	3,334	*DLX1*	Split hand and foot malformation 5	7
2q31.1	176,989,434∓177,009,843	20,410	*HOXD8, HOXD-AS2, LOC401021*	Synpolydactyly	7
7q11.21	62,501,593∓62,506,726	5,134	*-*	-	7
8p11.1	43,371,449∓43,383,206	11,758	*-*	-	7
14q32.2	101,290,932∓101,301,680	10,749	*MEG3*	MEG3/NA	7
19q13.43	57,349,301∓57,351,077	1,777	*ZIM2, PEG3*	PEG3/NA, PEG3_ICRegion/NA, ZIM2/NA	7
Xp11.23	48,324,187∓48,340,068	15,882	*SLC38A5, FTSJ1*	-	7
Xq13.1	70,347,097∓70,352,509	5,413	*MED12*	Opitz-Kaveggia	7

**Table 2:** Extents of chromosomal aberrations in 9 teratomas on array comparative genomic hybridization (aCGH) analysis

Cytoband	Tera-1	Tera-2	Tera-3	Tera-4	Tera-5	Tera-6	Tera-7	Tera-8	Tera-9	Average
2q31.1	0.276	0.239	0.676	0.414	0.176	0.333	0.160	0.216	0.238	0.303
11q13.2	1.071	0.445	1.251	0.919	0.710	0.382	0.415	0.552	0.416	0.685
Xq28	0.356	0.166	0.432	0.614	0.359	0.318	0.238	0.236	0.309	0.336
10q21.3	0.865	0.220	1.348	1.339	0.720	0.272	-	0.593	0.244	0.700
14q32.2	0.568	-	0.786	1.237	0.936	0.272	0.181	0.295	0.226	0.563
20q13.32	0.419	-	0.556	0.727	0.336	0.224	0.241	0.423	0.248	0.397
Xq13.1	0.377	-	0.483	1.664	0.295	0.357	0.161	0.281	0.196	0.477
Xq28	0.356	0.166	0.432	0.614	0.359	-	0.238	0.236	0.309	0.339
2q31.1	0.276	-	0.836	0.414	0.176	0.415	0.276	0.462	0.344	0.400
2q31.1	0.276	0.239	0.676	-	0.176	0.333	0.160	0.216	0.238	0.289
7q11.21	0.210	-	0.455	-	0.255	0.378	0.221	0.314	0.404	0.320
8p11.1	1.638	-	1.989	-	1.315	0.309	0.297	0.794	0.572	0.988
14q32.2	0.568	-	0.786	1.237	-	0.272	0.181	0.295	0.226	0.509
19q13.43	0.188	-	1.253	1.111	0.799	0.531	-	0.620	0.460	0.709
Xp11.23	0.622	-	1.132	1.578	0.429	0.219	-	0.599	0.370	0.707
Xq13.1	0.377	-	0.483	-	0.295	0.422	0.161	0.281	0.196	0.316

Amplifications of *EGR2* on 10q21.3 (average log2 ratio = 0.700) and a 499 bp segment of chromosome 14q32.2 (average log2 ratio = 0.563), *GNASAS* and *GNAS* on 20q13.32 (average log2 ratio = 0.397), *BCYRN1* and *ZMYM3* on Xq13.1 (average log2 ratio = 0.477), and *FLNA* and *EMD* on Xq28 (average log2 ratio = 0.339) were noted in most (8/9) teratomas (Tables 1 and 2). Chromosomal aberrations on aCGH analysis common to all nine teratomas are also listed in Tables 1 and 2.

Chromosomal aberrations on aCGH analysis common to four to six teratomas are listed in Suplemental Table 1. No specific aberration was noted in two bilateral mature cystic teratomas, right and left Tera-9 (data not shown).

### Immunohistochemistry

**Fig. 5:**
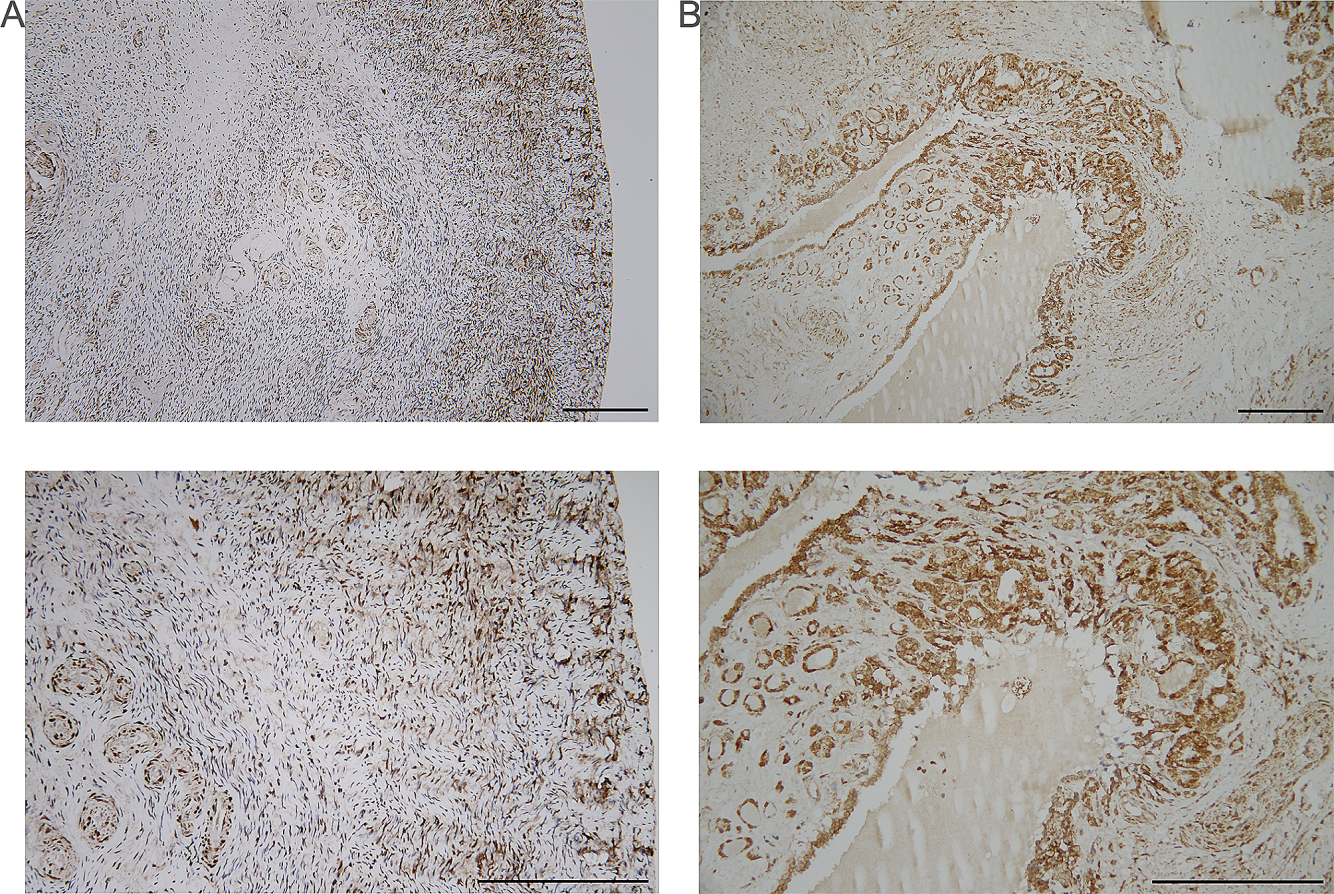
Immunohistochemical analysis of NDUFV1 staining. Light staining is present in the stroma of normal ovary (**A**) and intense staining is present in the parenchyma of Tera-2 (**B**). Upper panel is 100X and lower panel is 200X. Scale bar: 200 μm

Eight or more points had positive immunoreactive score. Semi-quantitative assessment of NDUFV1 staining by pathologists yielded negative scores of 2 × 2 = 4 (2 in intensity, 2 in proportion of the positive cells: 10–50%) for the stroma of normal ovary (Fig. 5A) and positive scores of 3 × 2 = 12 (3 in intensity, 4 in proportion of the positive cells: >80%) for the parenchyma of mature cystic teratoma of the ovary, Tera-2 (Fig. 5B).

## Discussion

Only two previous studies have used comparative genomic hybridization technique for detecting chromosomal aberrations in ovaian teratoma and none have used aCGH analysis. One study reported a case of mature ovarian teratoma with clonal chromosomal alterations including monosomies of chromosomes 6, 14, 16, and 21; trisomies of chromosomes 14 and 21; and deletions of Xq, 5p, 16p, and 17p [18]. Similar to trisomies of chromosomes 14, amplifications of a 499 bp segment of chromosome 14q32.2 (average log2 ratio = 0.563) were noted in most (8/9) teratomas in this study (Tables 1 and 2). Another study on benign cystic teratoma reported del(3q), add(4q), del(12q), and del(16p) in one case and del(6p), add(13q), and del(16p) in another case with bilateral tumors [19]. No such findings were noted in this study.

On aCGH analysis, comprised of 60,000 oligonucleotide probes at a genome-wide resolution of approximately 0.1 Mb, we found previously undescribed aberrations. In all cases, multiple unbalanced chromosomal aberrations were detected. It is possible to identify candidate genes for factors controlling spontaneous ovarian teratocarcinogenesis through aCGH analysis of ovarian teratomas. In an early study, Schneider et al. reported losses of 1p, 4q, and 6q and gains of 1q and 20q in 51 childhood germ cell tumors with comparative genomic hybridization [20]. Chromosomal gain of 12p is characteristic of germ cell tumors in adult patients [21]. However, only amplifications of *CLCNKA* and *CLCNKB* on 1p36.13 were noted in most (6/9) teratomas in this study (Supplemental Table 1). These results suggested that the basic characteristics of ovarian benign teratomas differ from those of germ cell tumors. Amiel et al. reported only two of nine cases with unbalanced chromosomal aberrations [19]. Deletion of 16p was observed in two cases and deletion of 6p was observed in one case [19]. None of these unbalanced chromosomal aberrations were observed in our ovarian mature cystic teratomas. This may be due to differences in ethnicity or reference gDNAs, or to higher resolution aCGH.

**Fig. 6:**
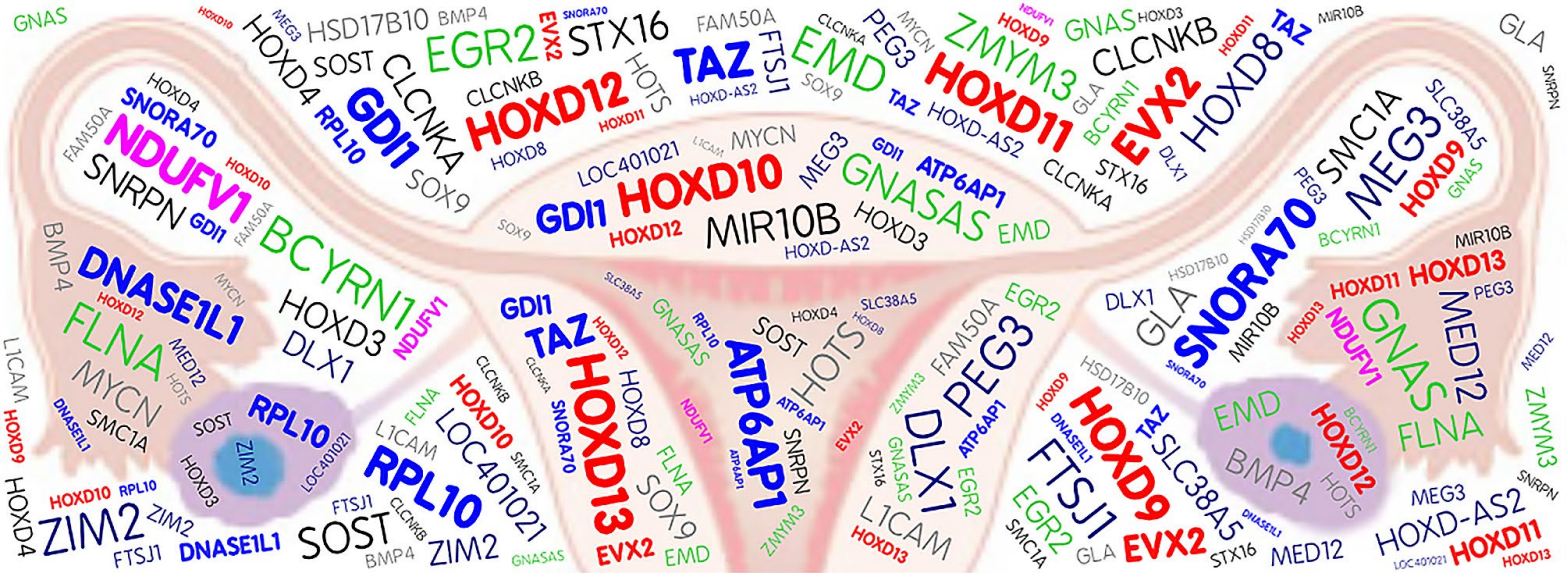
Word cloud artwork illustrates the important genes in ovarian mature cystic teratomas identified on aCGH. Amplifications of *EVX2* and *HOXD9-HOXD13* on 2q31.1 (red), *NDUFV1* on 11q13.2 (pink), and *RPL10, SNORA70, DNASE1L1, TAZ, ATP6AP1*, and *GDI1* on Xq28 (light blue) were found in all nine mature cystic teratomas. The chromosomal aberrations on aCGH analysis are present in eight (green), seven (dark blue), six (black), and five (gray) mature cystic teratomas of the ovary, respectively

*MYC* is a well-characterized human oncogene and common reprogramming factor for generating induced pluripotent stem cells [22]. Amplification of *MYCN* on 2p24.3 was noted in five teratomas in this study (Suplemental Table 1, shown gray in Fig. 6). Amplifications on chromosomes 1, 8, 12, 17 and X are the most common karyotypic abnormalities detected in pluripotent stem cells [22]. Amplifications on chromosomes 1p36.13, 8p11.1, 17q21.31, 17q24.3, Xp11.22, Xp11.23, Xp22.33, Xq13.1, Xq22.1, and Xq28 were noted in most teratomas in this study (Suplemental Table 1).

In addition, amplification of *NDUFV1* on a 2.8 kb segment of 11q13.2 was identified in all nine mature cystic teratomas (Fig. 3; Table 1, shown pink in Fig. 6). *NDUFV1* gene encodes a 51-kDa subunit of mitochondrial complex I, (NADH dehydrogenase [23] flavoprotein 1) NDUFV1, also known as UQOR1 [23]. Schuelke et al. (1999) detected mutations in the *NDUFV1* gene in patients with isolated complex I deficiency, nuclear type 4 [24]. *NDUFV1* is a putative developmental/neuropsychiatric susceptibility gene [25] and *NDUFV1* transcript has been shown to be overexpressed in blastocysts derived from superovulated heifers [26]. *NDUFV1* RNA, but not protein, expression has been detected in ovarian stroma cells in the Human Protein Atlas (www.proteinatlas.org) for normal and cancer tissues based on antibody proteomics [27]. The results of this study regarding absent (negative) NDUFV1 protein expression in the stroma of normal ovary (Fig. 5A) are consistent with those of a previous study [27]. Increased intact (positive) NDUFV1 protein expression of mature cystic teratomas of the ovary (Fig. 5B) indicated that NDUFV1 is a factor in induced differentiation.

In addition, amplifications of *RPL10, SNORA70, DNASE1L1, TAZ, ATP6AP1*, and *GDI1* on a 44.5 kb segment of Xq28 were identified in all nine mature cystic teratomas in this study (Fig. 4; Table 1, shown blue in Fig. 6). Vandewalle et al. (2009) observed 0.3-Mb copy number gain at chromosome Xq28 that included 18 annotated genes, of which RPL10, ATP6AP1, and GDI1 are expressed in the brain [28]. *GDI1* encodes GDP dissociation inhibitor 1, which plays an essential role in vesicle transport by slowing the rate of dissociation of GDP in the GDP-GTP exchange reaction of members of the rab family [29]. Vandewalle et al. (2009) considered *GDI1* the most likely candidate gene in the chromosome Xq28 duplication syndrome region [28]. Mutations in *GDI1* are responsible for X-linked non-specific mental retardation [30]. Dorus et al. (2004) demonstrated that GDI displays significantly higher rates of protein evolution in primates than in rodents and suggested that it plays a role in nervous system development [31]. *RPL10* (ribosomal protein L10 gene) encodes RPL10 protein, which is a constituent of the large subunit (60 S) of the ribosome [32]. Missense mutations in *RPL10* suggest susceptibility to X-linked autism 5 [33] and X-linked syndromic intellectual developmental disorder 35 [34]. De Keersmaecker et al. (2013) identified somatic mutations in *RPL10* in pediatric T-cell acute lymphoblastic leukemia [35]. RPL10 has also been suggested to drive oncogenic processes in the ovaries [36, 37]. *ATP6AP1* encodes the accessory S1 subunit (Ac45) of the enzyme V-type proton ATPase [38]. Jansen et al. (2016) identified hemizygous missense mutations in the *ATP6AP1* gene in males with immunodeficiency-47, also known as congenital disorder of glycosylation (CDG2S) [39]. This protein may also play a role in early development because mouse Atp6ap1 knockout embryonic stem cells do not give rise to viable embryos [40]. *SNORA70* (small nucleolar RNA, H/ACA Box 70), which resides (or is embedded) in the fifth intron of *RPL10*, is a non-coding RNA (ncRNA) molecule which functions in the biogenesis (modification) of other small nuclear RNAs (snRNAs) [41]. SNORA70 has oncogenic function in osteosarcomas [42]. *DNASE1L1* (deoxyribonuclease 1 Like 1) encodes a member of the deoxyribonuclease family and the protein shows high sequence similarity with lysosomal DNase I [43, 44]. DNA degradation is critical to healthy organism development and survival [45]. Its association with Pompe disease is controversial and with DNase1L1 is unclear [45]. *TAZ* encodes tafazzin, a transacylase essential for cardiolipin formation and central to the etiology of Barth syndrome, also known as 3-methyglutaconic aciduria type II [46]. Tafazzin promotes the tumorigenicity of cervical cancer cells and inhibits apoptosis [47]. Significant expression of tafazzin (TAZ) protein has been observed in colon cancer cells [48]. Further research is necessary to better understand the roles of these genes in the key mechanism for inducing differentiation during teratoma development.

The most interesting findings of this study are amplifications of *EVX2, HOXD13, HOXD12, HOXD11, HOXD10*, and *HOXD9* on a 41.6 kb segment of 2q31.1 in all nine mature cystic teratomas (Fig. 2, Table 1, shown red in Fig. 6). The *HOXD9-HOXD13* genes belong to a member of the *HOXD* cluster of homeobox genes that encode transcription factors involved in limb development [49] by the confined expression of SHH (Sonic hedgehog) at the posterior margin of developing early limb buds [50]. *EVX2* gene encodes a homeobox transcription factor that is related to the protein encoded by the Drosophila even-skipped (eve) gene, a member of the pair-rule class of segmentation genes [51]. *EVX2* and *HOXD9-HOXD13* are essential for proper development of the appendicular skeletons [52]. A 117 kb microdeletion at the 5’ end of the *HOXD* gene cluster, which includes *EVX2* and *HOXD9-HOXD13* genes, causes synpolydactyly [53]. *HOXD10* and *HOXD13* gene expressions have been found to be altered in primary breast cancers with respect to normal breast tissue, switching from off to on [54]. HOXD9 promotes epithelial-mesenchymal transition and metastasis in hepatocellular carcinoma [55] and colorectal carcinoma [56]. *HOXD10* is suppressed in colon adenocarcinoma cells [57]. Colon carcinomas show an overexpression of *HOXD10* which distinguishes colonic stem cells from colon carcinoma cells [58]. Woo et al. (2010) found that a 1.8 kb region between *HOXD11* and *HOXD12* has an activator function in the differentiation of human embryonic stem cells into mesenchymal stem cells (MSCs) and then into osteoblasts [59]. In humans, mutations in *HOXD13* cause dominantly inherited limb malformation synpolydactyly (SPD) [60]. Although no specific function of *HOXD* family genes in teratomas has been reported in humans, due to the relationship between these genes and differentiation in chondrogenesis and osteogenesis, we speculate that amplifications in *HOXD* family genes are associated with bone differentiation in teratomas. Only one previous study on teratocarcinoma embryoid bodies has suggested a possible role for *HOXD12* in establishing extraembryonic endoderm lineage in mice [61].

Teratoma, means strange tumor, as it contains tissues found in other parts of the body. Characteristic elements include skin, hair, fat, teeth, and bone [62]. It is interesting to understand the mechanisms involved in the development of so many different types of tissues in this tumor. From our previous report, *DUSP5* and *PHLDA1* mutations in mature cystic teratomas of the ovary identified on whole‑exome sequencing may explain teratoma characteristics in terms of osteogenic differentiation and hair growth [17]. Some intrinsic factors may also be involved, such as *HOXD* genes which are associated with bone development. In this study, *HODX9* (which affects cartilage formation [63]) to *HOXD13* (which affects the ossification of mature bone [64]) were amplified in all our teratoma samples. This may be the reason why the bone component is so common in teratomas and used for preoperative diagnosis. This is the first study on humans to support previous findings of chondrogenesis and osteogenesis on mouse model [63, 64]. Here, we provide new insights into mature cystic teratomas of the ovary, which increase our understanding of teratoma polydermal differentiation.

## Conclusions

In summary, amplifications of *EVX2, HOXD13, HOXD12, HOXD11, HOXD10*, and *HOXD9* on 2q31.1, *NDUFV1* on 11q13.2, and *RPL10, SNORA70, DNASE1L1, TAZ, ATP6AP1*, and *GDI1* on Xq28 were found in all nine mature cystic teratomas in this study. Our results indicated that amplifications of these genes play an important etiological role in teratoma formation. Moreover, amplifications of *EVX2* and *HOXD9-HOXD13* on 2q31.1 may help to explain the characteristics of teratomas in chondrogenesis and osteogenesis.

### Electronic supplementary material

Below is the link to the electronic supplementary material.

Supplementary Material 1: Histological characteristics of mature cystic teratomas of the ovary (hematoxylin and eosin, H&E) with ruler. Scale bar: 200 μm

Supplementary Material 2: Chromosomal aberrations on array comparative genomic hybridization (aCGH) analysis present 4 to 6 teratomas

## Data Availability

The original contributions presented in the study are included in the article. Further inquiries can be directed to the corresponding author.

## Abbreviations

CGH: comparative genomic hybridization
DNASE1L1: deoxyribonuclease 1 Like 1
DNA: deoxyribonucleic acid
EVX2: Even-Skipped Homeobox 2
GDI1: GDP dissociation inhibitor 1
HOXD: homeobox D cluster
NDUFV1: NADH ubiquinone oxidoreductase complex I
RPL10: ribosomal protein L10
SNORA70: small nucleolar RNA, H/ACA Box 70
TAZ: Tafazzin
UV-VIS: Ultraviolet–visible
