# Supplementary material for: Amplifications of EVX2 and HOXD9-HOXD13 on 2q31 in mature cystic teratomas of the ovary identified by array comparative genomic hybridization may explain teratoma characteristics in chondrogenesis and osteogenesis
Source: J Ovarian Res. 2024 Jun 21;17:129. doi: 10.1186/s13048-024-01458-5 (PMC11193297; doi:10.1186/s13048-024-01458-5)
Supplement: Supplementary file 1 — Supplementary Material 1: Histological characteristics of mature cystic teratomas of the ovary (hematoxylin and eosin, H&E) with ruler. Scale bar: 200 μm [file 13048_2024_1458_MOESM1_ESM.docx]

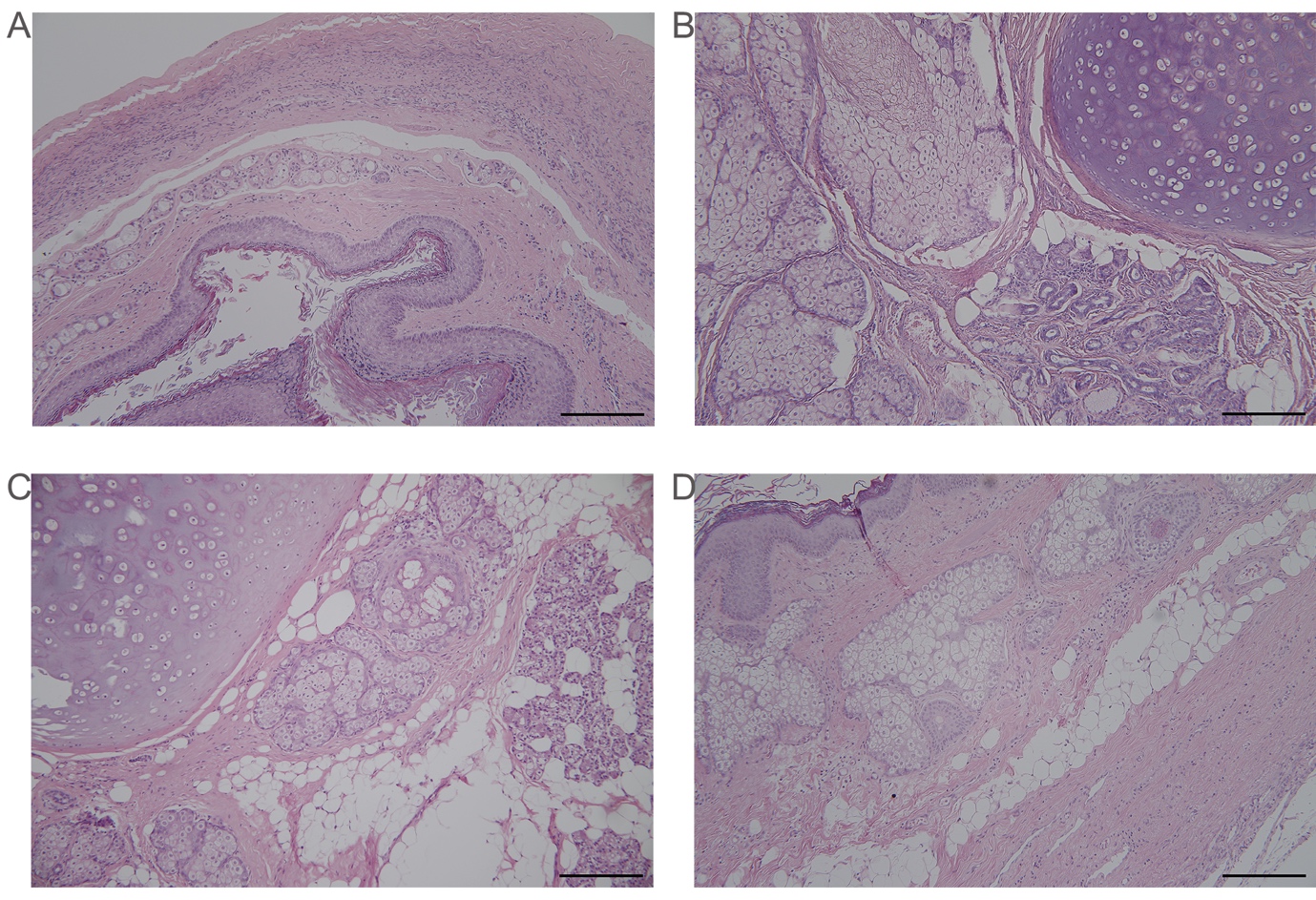


Additional file 1. Histological characteristics of mature cystic teratomas of the ovary (hematoxylin and eosin, H&E) with a ruler. Tera-6 (A), Tera-7 (B), Tera-8 (C), and Right Tera-9 (D) are present in 100X. Scale bar: 200 μm.
